# Enhancing epitope of PEDV spike protein

**DOI:** 10.3389/fmicb.2022.933249

**Published:** 2022-07-22

**Authors:** Techit Thavorasak, Monrat Chulanetra, Kittirat Glab-ampai, Kodchakorn Mahasongkram, Nawannaporn Sae-lim, Karsidete Teeranitayatarn, Thaweesak Songserm, Rungrueang Yodsheewan, Dachrit Nilubol, Wanpen Chaicumpa, Nitat Sookrung

**Affiliations:** ^1^Graduate Program in Immunology, Department of Immunology, Faculty of Medicine Siriraj Hospital, Mahidol University, Bangkok, Thailand; ^2^Center of Research Excellence in Therapeutic Proteins and Antibody Engineering, Department of Parasitology, Faculty of Medicine Siriraj Hospital, Mahidol University, Bangkok, Thailand; ^3^MORENA Solution Company, Bangkok, Thailand; ^4^Department of Veterinary Pathology, Faculty of Veterinary Medicine, Kasetsart University, Bangkok, Thailand; ^5^Department of Veterinary Microbiology, Faculty of Veterinary Science, Chulalongkorn University, Bangkok, Thailand; ^6^Swine Viral Evolution and Vaccine Development Research Unit, Chulalongkorn University, Bangkok, Thailand; ^7^Biomedical Research Incubation Unit, Department of Research, Faculty of Medicine Siriraj Hospital, Mahidol University, Bangkok, Thailand

**Keywords:** antibody-dependent enhancement, enhancing epitope, phage display technology, phage panning, phage mimotope, porcine epidemic diarrhea, porcine epidemic diarrhea virus, spike protein

## Abstract

Porcine epidemic diarrhea virus (PEDV) is the causative agent of a highly contagious enteric disease of pigs characterized by diarrhea, vomiting, and severe dehydration. PEDV infects pigs of all ages, but neonatal pigs during the first week of life are highly susceptible; the mortality rates among newborn piglets may reach 80–100%. Thus, PEDV is regarded as one of the most devastating pig viruses that cause huge economic damage to pig industries worldwide. Vaccination of sows and gilts at the pre-fertilization or pre-farrowing stage is a good strategy for the protection of suckling piglets against PEDV through the acquisition of the lactating immunity. However, vaccination of the mother pigs for inducing a high level of virus-neutralizing antibodies is complicated with unstandardized immunization protocol and unreliable outcomes. Besides, the vaccine may also induce enhancing antibodies that promote virus entry and replication, so-called antibody-dependent enhancement (ADE), which aggravates the disease upon new virus exposure. Recognition of the virus epitope that induces the production of the enhancing antibodies is an existential necessity for safe and effective PEDV vaccine design. In this study, the enhancing epitope of the PEDV spike (S) protein was revealed for the first time, by using phage display technology and mouse monoclonal antibody (mAbG3) that bound to the PEDV S1 subunit of the S protein and enhanced PEDV entry into permissive Vero cells that lack Fc receptor. The phages displaying mAbG3-bound peptides derived from the phage library by panning with the mAbG3 matched with several regions in the S1-0 sub-domain of the PEDV S1 subunit, indicating that the epitope is discontinuous (conformational). The mAbG3-bound phage sequence also matched with a linear sequence of the S1-BCD sub-domains. Immunological assays verified the phage mimotope results. Although the molecular mechanism of ADE caused by the mAbG3 *via* binding to the newly identified S1 enhancing epitope awaits investigation, the data obtained from this study are helpful and useful in designing a safe and effective PEDV protein subunit/DNA vaccine devoid of the enhancing epitope.

## Introduction

Porcine epidemic diarrhea virus (PEDV) is an etiologic agent of a highly contagious disease of pigs named porcine epidemic diarrhea (PED) which is characterized by acute diarrhea, vomiting, and severe dehydration (Lee, 2015). The virus can infect pigs of all ages, but the disease is highly fatal among neonatal pigs during the first 7–10 days of lives and the mortality rate may reach up to 80–100% (Pensaert and de Bouck, 1978; Lee, 2015; Jung et al., 2020). PEDV-infected neonatal piglet manifests acute viremia and severe atrophic enteritis (mainly jejunum and ileum), with increased pro-inflammatory and innate immune responses (Annamalai et al., 2015; Jung et al., 2018). The PEDV is shed in the watery stool and nasal discharge of the infected pigs and spreads further (Jung et al., 2020). Pigs are more tolerable to PED as they grow older, but asymptomatically infected older pigs on a farm may serve as the virus reservoirs for the subsequent outbreaks (Wang et al., 2019). PED was first recognized in 1971 in England; the disease subsequently spread to other European countries, North and South Americas, and Asia (Lee, 2015; Jung et al., 2020). The virus is now regarded as one of the most devastating pig viruses causing severe economic damage to pig industries worldwide.

Porcine epidemic diarrhea virus is a large, enveloped, plus-sense RNA virus of the genus *Alphacoronavirus*, family *Coronaviridae*, and order *Nidovirales* (Lee, 2015; Jung et al., 2020). The PEDV genome is approximately 28 kb long and consists of a 5′-untranslated region (UTR) with a cap, followed by at least seven open reading frames (ORF1a, ORF1b, and ORFs 2–6), and a 3′-polyadenylated tail (Lee, 2015). The ORFs 1a and 1b that occupy two-thirds of the genome at the 5′ end code for multifunctional polyproteins (pp) la and pp1ab, which are further post-translationally cleaved by the *cis* and *trans* proteases of the virus to generate 16 functionally different non-structural proteins (nsps), nsp1–16. The mature nsps form replicase/transcriptase complex (RTC) to generate full-length genomic RNA and sub-genomic (sg) mRNAs from the remaining ORFs that constitute one-third of the genome at the 3′ end. The sg mRNAs are translated into four structural proteins, including spike (S) protein which is post-translationally glycosylated (150–220 kDa), membrane (M) protein (20–30 kDa), envelope (E) protein (7 kDa), and nucleocapsid (N) protein (58 kDa), and accessory gene ORF3 (Li et al., 2020).

The PEDV uses the S1 subunit located at the N-terminal end of the surface-exposed spike (S) glycoprotein to bind to the receptors on the pig enterocyte, followed by virus–host membrane fusion mediated by the S2 subunit at the C-terminal end of the S ectodomain to release the virus genome into the cytosol for further replication (Lee, 2015). The S1 subunit is composed of the leader peptide sequence (residues 1–18) and sub-domains 0 (residues 19–219), A (residues 220–509), B (residues 510–639), and CD (residues 640–728); the S2 subunit starts at residue 729 and extends up to the C-terminal end (Li et al., 2017). Heterogeneity in the genomic sequences of the PEDV S protein has been reported and used for the classification of isolated strains (Lin et al., 2016). Currently, PEDV isolates are classified into two genotypes (GI and GII) and five sub-genotypes (GIa, GIb, GIIa, GIIb, and GIIc; Guo et al., 2019). The GI is represented by classical PEDV strain CV777 and the CV777-like strains that have emerged since the 1970s, while the GII includes highly virulent strains (field isolates) detected globally after 2010 (Lin et al., 2016). The emerging PEDV strains are also divided into two major groups based on the S gene features and their virulence in young pigs, i.e., the highly virulent non-S INDEL strains (insertions and deletions) that cause pandemic PED outbreaks worldwide and the less pathogenic (cause lower mortality) S INDEL strains [the N-terminal region of the S protein of these strains has an amino acid insertion (residues 161–162) and two deletions (residues 59–62 and 140) compared with those of the highly virulent PEDV strains] (Wang et al., 2014; Lin et al., 2016).

Vaccination against PEDV is a useful strategy to control the disease regardless of the type of immunogens used in the vaccine (killed, live, recombinant subunit, and DNA-vectored) and administration route (intramuscular versus oral; Won et al., 2020). However, the vaccine must be given in multiple, spaced doses to sows or gilts either at pre-fertilization or pre-farrowing stage to induce high levels of neutralizing antibodies (mainly directed toward the PEDV S protein to block the virus entry into the host cells) in colostrum and milk for conferring lactogenic immunity to the suckling newborns. The immunization protocols of the vaccines are complicated, and the protective efficacies among the passively immunized suckling piglets are inconsistent. Some studies obtained satisfactory results including reduced morbidity and mortality, shortened period of virus shedding in the feces, and quick recovery of daily weight gain, while other studies did not (Gerdts and Zakhartchouk, 2017). One of the major obstacles to vaccination against PEDV and other coronaviruses is the vaccine-induced antibody-dependent enhancement (ADE) of new infection (Wen et al., 2020; Xu et al., 2021; Yu et al., 2021). For safety issues, the vaccine should not contain epitopes that elicit enhancing antibodies (Xu et al., 2021; Yu et al., 2021). Several neutralizing B-cell epitopes of the PEDV spike protein have been identified (Thavorasak et al., 2022). On contrary, data on enhancing epitopes, which is the dark side of PEDV protein, are scarce, if there were any. In this study, a novel B-cell epitope of the PEDV S1 protein that enhanced the virus entry into the host cells is reported.

## Materials and methods

### Cells and virus

African green monkey kidney epithelial (Vero) cells were obtained from American Type Culture Collection (ATCC, Manassas, VA, United States). The cells were maintained in Dulbecco’s Modified Eagle’s Medium (DMEM; Thermo Fisher Scientific, Waltham, MA, United States) supplemented with 10% fetal bovine serum (FBS; Sigma-Aldrich, St. Louis, MO, United States), 2 mM l-alanyl-l-glutaminase dipeptide, 100 IU/ml penicillin, and 100 μg/ml streptomycin (Thermo Fisher Scientific).

Mouse hybridoma G3 secreting monoclonal antibody (mAbG3; IgG1-kappa isotype) that bound to the S1 protein of the PEDV was generated previously (Thavorasak et al., 2022). The cells were nurtured at 37°C in a humidified atmosphere containing 5% CO_2_ in an incubator in a serum-free medium [CD medium supplemented with 8 mM l-alanyl-l-glutaminase dipeptide, 100 IU/ml penicillin, and 100 μg/ml streptomycin (Thermo Fisher Scientific)].

The PEDV (GII variant, strain P70, and isolated from PEDV-infected piglet in Thailand) was propagated in the Vero cells cultured in virus maintaining medium [DMEM supplemented with 2 μg/ml of *N*-tosyl-l-phenylalanine chloromethyl ketone (TPCK)-treated trypsin (Sigma-Aldrich)]. Vero cell monolayers at ~80–100% confluent growth in the T175 culture flask were rinsed with sterile phosphate-buffered saline, pH 7.4 (PBS); the PEDV was added to the cells at MOI 0.001 and incubated at 37°C in a humidified atmosphere containing 5% CO_2_ in an incubator for 1 h. The fluid was removed, the cells were rinsed twice with PBS, replenished with the virus maintaining medium, and incubated at 37°C in humidified 5% CO_2_ atmosphere in an incubator. When the cytopathic effect (CPE; syncytial formation) reached the maximum level (approximately 48 h), the culture supernatant containing the PEDV was harvested, and centrifuged at 4500 × *g* and 4°C for 30 min to remove cells and debris. The clarified culture supernatant was filtered through a 0.2 μm Acrodisc^®^ syringe filter (Pall, Port Washington, NY, United States) and stored in small portions at −80°C until further use. The virus titer was determined by plaque-forming assay (PFA) and reported as plaque-forming units (pfu)/ml.

### Preparation of mAbG3

For the production of mouse monoclonal antibody G3 (mAbG3), log phase grown hybridoma G3 cells were seeded into a serum-free medium (CD medium, Thermo Fisher Scientific) at 3 × 10^5^ cells/ml and incubated further at 37°C in humidified 5% CO_2_ atmosphere in an incubator for 96 h. The culture supernatant containing the mAbG3 was harvested, and centrifuged at 4500 × *g* and 4°C for 30 min to remove the cells and cell debris. The clarified supernatant was subjected to Hitrap protein G HP (Cytiva, Uppsala, Sweden) chromatography for the purification of the mAbG3. The protein content of the purified mAbG3 was quantified using the BCA assay (Thermo Fisher Scientific).

### Virus neutralization assay

The neutralization assay was performed as described previously (Thavorasak et al., 2022). Purified mAbG3 (5–40 μg in 125 μl of PBS) was mixed with 100 pfu of PEDV P70 and incubated at 37°C for 1 h. Immune serum to PEDV S1 subunit (1:100), mAbA3 (Thavorasak et al., 2022), isotype-matched control mAb (IgG1-kappa; BioLegend, San Diego, CA, United States), and culture medium alone were included in the experiments as controls. The mixtures were then added to the confluent monolayers of Vero cells in a 24-well tissue culture plate. After 1 h of incubation, the supernatant was discarded, and the cells were rinsed for two times to remove the unbound virus. Carboxymethyl cellulose (2% CMC in DMEM supplemented with 2 μg of TPCK-treated trypsin) was added. At 48 h post-infection, the cells were fixed with 10% formalin, kept at room temperature (25 ± 2°C) for 1 h, and stained with 1% crystal violet in 10% ethanol. The CPE (syncytial formation) was counted under a light microscope at ×40 magnification. The percentage of mAbG3-mediated PEDV neutralization/enhancement was calculated: [1-(Average CPE count of test/Average CPE count of non-neutralization control)] × 100.

### Real-time (quantitative) RT-PCR (qRT-PCR)

Alternatively, the PEDV RNAs recovered from the treated infected cells were determined by qRT-PCR. The virus neutralization assay was performed as described above with a slight modification. The DMEM supplemented with 2 μg of TPCK-treated trypsin was used instead of 2% CMC in DMEM supplemented with 2 μg of TPCK-treated trypsin. After 6 h post-infection, total RNA from the mAbG3/control-treated infected cells was extracted separately using TRIzol^®^ reagent (Thermo Fisher Scientific). The amount of viral RNA was quantified by qRT-PCR using a one-step brilliant III SYBR green RT-qPCR master mix (Agilent Technologies, Santa Clara, CA, United States). The qRT-PCR primers for the PEDV nucleocapsid (N) gene and house-keeping gene control are listed in Supplementary Table S1. The PCR reaction mixture consisted of 6 μl of nuclease-free water, 10 μl of 2× SYBR Green QPCR master mix, 0.4 μl of 10 μM of each specific primer, 0.2 μl of 100 mM dithiothreitol (DTT), 1 μl of RT/RNase block, and 2 μl of RNA template. The conditions for PCR thermal cycles were 50°C for 10 min, 95°C for 3 min, 40 cycles at 95°C for 5 s, and 58°C for 10s, followed by melt curve analysis protocol. The copy numbers of viruses were calculated from Cq values and compared.

### Production of S1 subunit of PEDV spike protein and the S1 subunit truncated polypeptides

The full-length S1 subunit of the PEDV was prepared as described previously (Thavorasak et al., 2022). For preparing the S1 subunit truncated polypeptides, i.e., S1-0 (residues M1-V217), S1-A (residues T218-N508), and S1-BCD (residues D509-T728; Li et al., 2017), total RNA was isolated from culture supernatant containing PEDV using TRIzol reagent (Thermo Fisher Scientific). The isolated RNA was subjected to RevertAid First Strand cDNA Synthesis Kit using a reverse primer specific to the PEDV E gene for synthesizing the cDNA. The cDNA was then used as a template for the amplification of DNA sequences coding for the S1-0, S1-A, and S1-BCD polypeptides using specific primers listed in Supplementary Table S2. The amplified DNA sequences were ligated separately to pJET1.2 plasmids, and the recombinant plasmids were transformed into DH5α *E. coli*. The transformed DH5α *E. coli* colonies with the respective DNA inserts were cultured in Luria-Bertani (LB) broth supplemented with 100 μg/ml ampicillin (LB-A) at 37°C with shaking aeration (250 rpm) and incubated overnight. The recombinant plasmids were then extracted from the DH5α *E. coli* and subcloned into pTriEx1.1 Hygro DNA (Merck KGaA, Darmstadt, Germany) *via Bam*HI and *Xho*I restriction sites. The ligated plasmids were transformed into NiCo21 (DE3) *E. coli*. Transformed NiCo21 (DE3) *E. coli* clones that carried the correct DNA inserted sequences (verified by Sanger sequencing) were grown in LB-A broth at 37°C with shaking aeration until OD at 600 nm was 0.4–0.5. Isopropyl β-d-1-thiogalactopyranoside (IPTG) was added to individual cultures to 0.1 mM final concentration, and the cultures were incubated further for 3 h. The 8× His-tagged recombinant polypeptides were purified from the bacterial homogenates by using Ni-NTA affinity resin (Thermo Fisher Scientific) under denaturing conditions.

### SDS-PAGE and Western blot analysis

One microgram each of the full-length recombinant S1 (rS1), S1-0, S1-A, and S1-BCD were prepared in protein loading buffer and subjected to 12% sodium dodecyl sulfate-polyacrylamide gel electrophoresis (SDS-PAGE). The separated proteins were blotted onto the nitrocellulose membrane (NC). The empty sites of blotted NC were blocked with 5% skim milk in Tris-buffered saline containing 0.1% Tween-20 (TBST) at room temperature for 1 h. After washing with TBST, the NC was immersed into a solution of either mouse anti-6× His antibody or purified mAbG3 solution (5 μg in 6 ml TBST) and kept at room temperature for 1 h. The membrane was washed with the TBST and placed in a solution of goat anti-mouse Ig-alkaline phosphatase (AP) conjugate (1:3,000; Southern Biotech, Birmingham, AL, United States) in TBST at room temperature for 1 h. After washing, BCIP/NBT substrate (KPL, Gaithersburg, MD, United States) was used for the visualization of the antigen–antibody reactive bands. The enzymatic reaction was stopped by rinsing the NC with distilled water.

### ELISAs for determining binding of mAbG3 to the S1 polypeptides

For indirect ELISA, individual wells of 96-well microplate (Nunc MaxiSorp^™^, Thermo Fisher Scientific) were coated with 1 μg of purified S1, S1-0, S1-A, and S1-BCD in 100 μl of PBS. The plate was kept at 4°C overnight. The supernatants were discarded, and the empty sites of the well surface were blocked with 1% BSA in PBS containing 0.1% Tween-20 (PBST) at 37°C for 1 h. One hundred microliters of PBST containing 1 μg of mAbG3 was added to each antigen-coated well and incubated at 37°C for 1 h. The wells were washed with PBST, added 100 μl of goat anti-mouse Ig-horseradish peroxidase conjugate (HRP; 1:5,000; Southern Biotech) in PBST, and kept at 37°C for 1 h. The fluids were discarded; all wells were washed with PBST, added 100 μl of ABTS substrate (KPL) individually, and the plate was kept at room temperature in darkness for 30 min. The OD of the content of each well was measured at 405 nm against blank (reactants without antibody) by spectrophotometry (BioTek, Winooski, VT, United States). Mouse anti-6× His antibody and PBS (instead of mAbG3) were included as positive and negative controls, respectively.

For dot ELISA, 1 μg of purified S1-0, S1-A, and S1-BCD were dotted separately onto the NC strip by using the Bio-Dot^®^ microfiltration apparatus (Bio-Rad). The membranes were dried for 30 min and blocked with 5% skim milk in TBST for 1 h at room temperature. After washing with TBST, 5 μg of mAbG3 in 6 ml of TBST was added and kept at room temperature for 1 h. Mouse anti-6× His antibody (1:1,000; Bio-Rad) and PBS were included as positive and negative controls, respectively. After washing, the membranes were immersed in goat anti-mouse Ig-alkaline phosphatase conjugate (1:3,000; Southern Biotech) in TBST at room temperature for 1 h. After washing, BCIP/NBT substrate (KPL) was used to visualize the reactive dots on membranes.

### Identification of phage mimotopes for determination of the PEDV S1 sub-domains bound by the mAbG3

To identify the region (sub-domains) of the PEDV S1 subunit that was bound by the mAbG3, Ph.D.^™^- 12 Phage Display Peptide Library (New England Biolab, Ipswich, MA, United States) was used for panning with the mAbG3. For this experiment, 1 μg of purified mAbG3 was added to a well of 96-well microplate (Nunc MaxiSorp^™^) and kept at 4°C overnight. The empty sites of the well surface were blocked with 1% BSA in TBST. After washing, a diluted phage library (~2 × 10^11^ peptide displaying phages in 100 μl of 0.5% BSA in TBST) was added to the mAbG3 coated well and incubated at room temperature for 1 h. The unbound phages were removed by washing thoroughly with TBST. The bound phages were eluted with 0.2 M glycine HCl (pH 2.2) for 10 min and immediately neutralized with 1 M Tris–HCl (pH 9.1). The phages were added to an early log phase grown ER2738 *E. coil* to amplify the phages and kept at 37°C for 4.5 h. The culture supernatant was harvested after centrifugation at 12,000× *g* and 4°C for 15 min The clear supernatant was mixed with 1/6 volume of 20% (w/v) PEG8000 and 2.5 M NaCl to precipitate and concentrate the phages. The concentrated phages were titrated on LB/IPTG/X-gal plates and used for the second- and third-round phage panning. The eluted phages of the third-round panning were diluted in LB broth (10^1^–10^4^), inoculated into mid-log phase grown ER2738 *E. coli*, mixed with prewarmed (45°C) top agar, and poured onto LB/IPTG/X-gal agar plates. The plates were incubated at 37°C for 16 h. Ten single blue colonies were randomly picked and inoculated into mid-log phase grown ER2738 *E. coli* for phage amplification. The *E. coli* culture supernatants were collected for phage DNA isolation and sequencing. The DNA sequences coding for the peptides displayed by the mAbG3-bound phages were deduced by using QIAGEN CLC workbench program (Version 20.0.4^1^; accessed on 11 March 2022). The phage mimotopes (M types) were then multiply aligned with the monomeric S1 sequence of PEDV GI classical strain CV777 and GII P70 strain by using Jalview software (Version 2.11.2.2; Waterhouse et al., 2009) to determine the region of the S1 subunit that matched with the mimotope sequences, i.e., the mAbG3 epitope.

### Peptide binding ELISA

To identify the enhancing epitope of the mAbG3 on the PEDV S1 subunit, peptide binding ELISA was performed. Five biotin-labeled 12 amino acid peptides of the PEDV S1 subunit that matched with the mAbG3-bound phage mimotopes and a control peptide were synthesized (Genscript, Piscataway, NJ, United States). Individual peptides were diluted in PBS to 10 μg/ml, and 100 μl of the diluted peptides were added to the separate wells (triplicate) of the streptavidin-coated microplate (Pierce^™^ Streptavidin Coated HighCapacity Plates; Thermo Fisher Scientific), and the plate was kept at 4°C overnight. The plate was washed three times with TBST, and the empty sites of the well surface were blocked with 5% skim milk in TBST. After 1 h at 37°C, the fluids were discarded. The wells were washed again, and 1 μg of purified mAbG3 in 100 μl of TBST was added to the individual wells. Buffer was included as a negative antibody control. The plate was incubated at 37°C for 1 h. After washing, 100 μl of HRP-labeled mouse IgG-kappa binding protein (Santa Cruz Biotechnology, Dallas, TX, United States; 1:1,000 in TBST) was added to each well, and the plate was incubated further at 37°C for 1 h. After washing, ABTS substrate (KPL; 100 μl) was added to each well, and the plate was kept in darkness at room temperature for 30 min. The OD of individual wells was measured at 405 nm (BioTek spectrometer).

### Multiple sequence alignment

Amino acid sequences of the S1 subunits of the PEDV strains that have been submitted to the National Center of Biotechnology Information (NCBI) database within the past 5 years, including PEDV SD2021 (GenBank: UJZ92254.1), PEDV TRS2021 (GenBank: UJZ92268.1), TW/PT01/2020 (GenBank: UOK15705.1), SC-YB73 (GenBank: QNL15619.1), XJ1904-34 (GenBank: UGN13709.1), PEDV-H18-Barcelona-Vic (GenBank: QKV43853.1), PEDV-2118-1-Orense-Covelas (GenBank: QKV43823.1), CT P10 (GenBank: QHB92364.1), EdoMex/205/2018 (GenBank: QQK84868.1); and 0100/5P (GenBank: UAJ21031.1), as well as CV777 (classical strain, GenBank: AEX92968.1), USA/Colorado/2013 (prototypic strain used for producing baculovirus-derived S subunit vaccine that caused severe PEDV enhancement in immunized piglets in a challenge study, Yu et al., 2021; GenBank: KF272920.1), and our P70 strain, were multiply aligned and compared.

### Localization of the mAbG3 epitope on the PEDV spike protein

To locate the S1 epitope that bound to mAbG3, a cryoelectron structure of PEDV spike protein (PDB: 6vv5; Kirchdoerfer et al., 2021) was subjected to PyMOL software (Version 4.6; The PyMOL Molecular Graphics System, Schrödinger LLC, New York, NY, United States) for generating the spike protein structure, and the locations of the mAbG3 epitope were mapped on the S protein structure.

### Statistical analysis

Statistical analysis of data was performed using GraphPad Prism software (Version 9.2; San Diego). Data were analyzed by using one-way ANOVA and were considered significantly different when *p* < 0.05.

## Results

### The mAbG3-mediated enhancement of PEDV infectivity instead of neutralization

**Figure 1:**
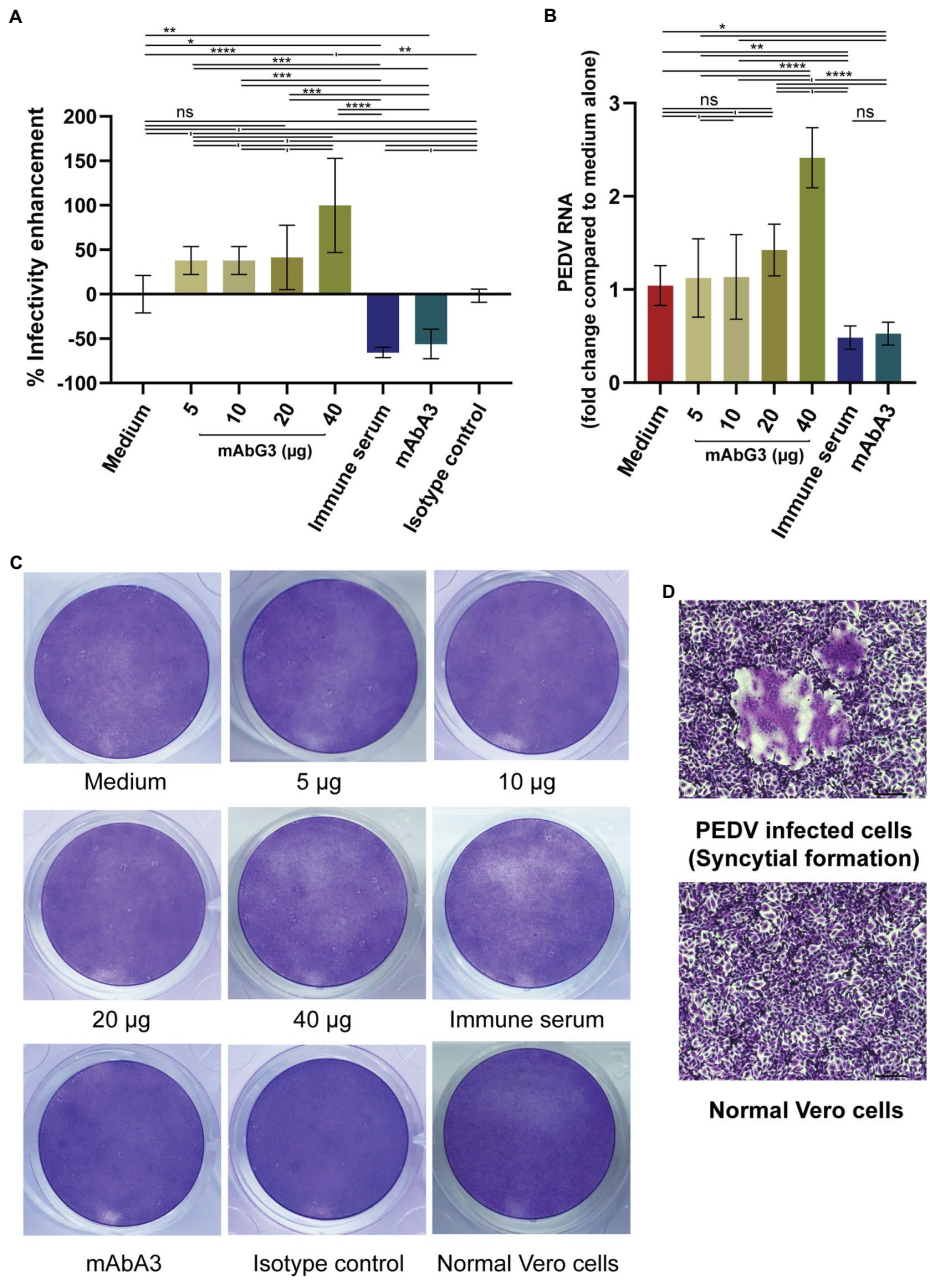
Enhancement of the PEDV infectivity mediated by the mAbG3. **(A)** The mAbG3 at the concentrations of 5, 10, 20, and 40 μg was mixed with 100 pfu of PEDV GII strain P70 and incubated for 1 h before adding to the confluent monolayers of Vero cells. Immune serum to PEDV S1 subunit (1:100) and mAbA3 (40 μg) served as positive neutralization controls, medium alone served as negative neutralization/enhancement control, and isotype-matched IgG1-kappa mAb served as irrelevant mAb control. After 1 h of incubation, the fluid in each well was removed; the cells were rinsed and covered with 2% CMC in DMEM supplemented with 2 μg/ml of TPCK-treated trypsin. Two days post-infection, cells were fixed, and the number of syncytial formations was counted and calculated to % enhancement of PEDV infectivity compared to the medium alone. Data of each bar graph are presented as the mean and standard deviation of the % mAbG3-mediated enhancement of PEDV infectivity from triplicate wells of each treatment. **(B)** Results of qRT- PCR for determination of PEDV RNA recovered from different treatment groups. Y-axis, fold changes of PEDV RNA in different treatment groups compared to medium alone; X-axis, various treatment groups. By one-way ANOVA: ns, not significantly different; ^*^, *p* < 0.05; ^**^, *p* < 0.01; ^***^, *p* < 0.001; ^****^, *p* < 0.0001. **(C)** Examples of the PEDV-mediated Vero cell CPE of different treatments. **(D)** Appearance of syncytial formation of PEDV-infected Vero cells (upper panel) compared with normal Vero cells (lower panel) at ×100 magnification of a light microscope.

About 5, 10, 20, and 40 μg concentrations of purified mAbG3 were mixed with PEDV GII field isolated P70 strain and incubated at 37°C for 1 h before adding to the confluent monolayers of Vero cells. After allowing the virus adsorption for 1 h, the fluids were removed, and the cells were rinsed two times before adding 2% CMC in 500 μl of DMEM supplemented with 2 μg/ml of TPCK-treated trypsin. Two days post-infection, the cells were fixed and stained with 10% formalin and 1% crystal violet in 10% ethanol, respectively. Mouse immune serum to PEDV S1 subunit (1:100), 40 μg of mAbA3 (Thavorasak et al., 2022), 40 μg of isotype-matched control mAb (IgG1-kappa), and culture medium alone were included in the experiments as controls. Cytopathic effect (CPE) was determined under light microscopy at ×40 magnification. It was found that instead of mediating neutralization of the PEDV infectivity, on contrary, the mAbG3 at 40 μg concentration significantly enhanced the PEDV infectivity by increasing CPE (the numbers of syncytia) compared to the medium (*p* < 0.0001) and the isotype control (*p* < 0.01), while the immune serum and the mAbA3 neutralized the PEDV infectivity (Figure 1A). The mAbG3 at 5, 10, and 20 μg concentrations showed a trend toward the enhancement of infectivity, but the CPE counts were not significantly different from the medium alone (*p* > 0.05). The results of qRT-PCR conformed to the CPE results (Figure 1B). Examples of the PEDV-mediated CPE in the Vero cell monolayer of different virus treatments are shown in Figure 1C. A comparison of the Vero syncytium to normal Vero cells is shown in Figure 1D. Three independent experiments were performed with reproducibility.

### Identification of tentative mAbG3 epitope by phage mimotope search and phage peptide alignment with the PEDV S1 linear sequence

**Figure 2:**
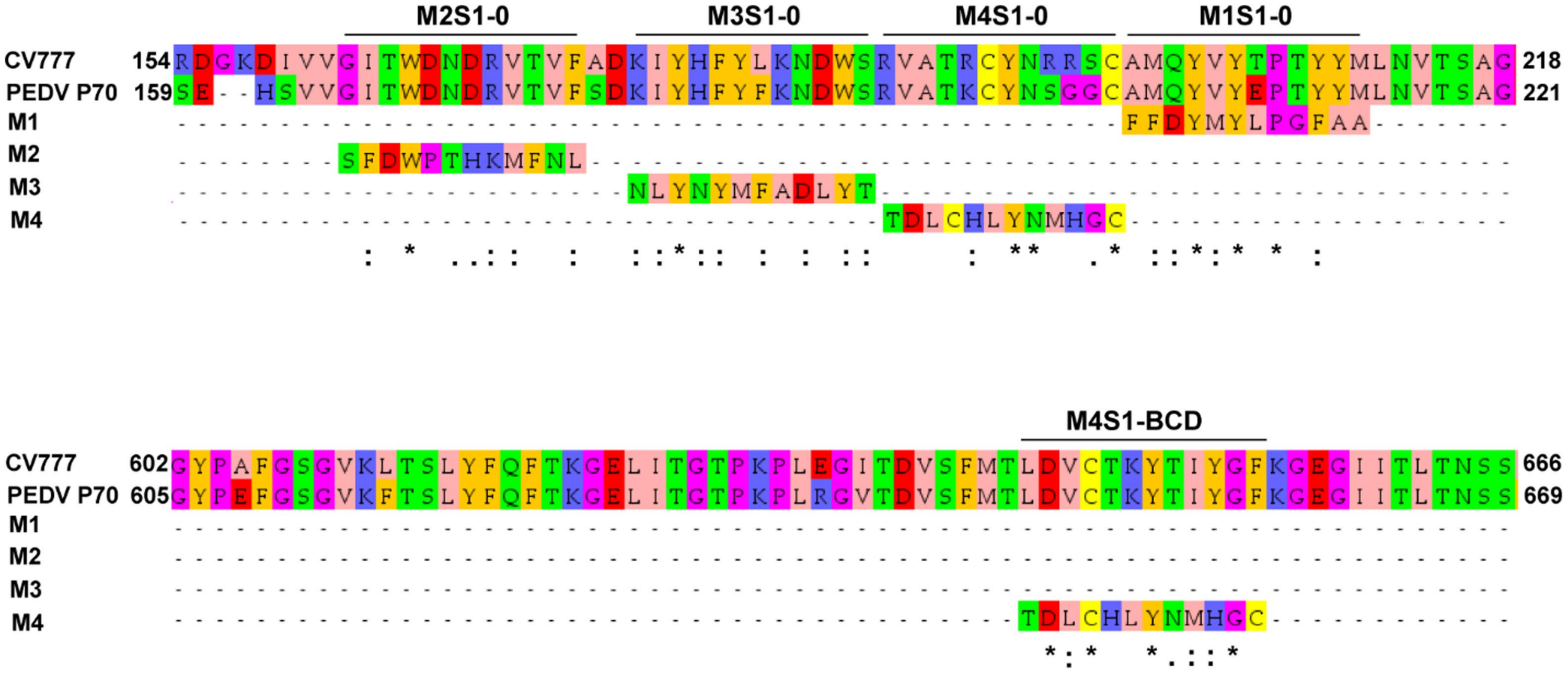
Linear amino acid sequences of S1-0 (residues 154–218/221) and S1-BCD (residues 602–666/669) sub-domains of S1 proteins of PEDV strains CV777 (GI; GenBank: AEX92968.1) and P70 (GII) showing regions matched with the phage mimotope types 1–4 (M1-M4), i.e., the S1-0 residues 165GITWDNDRVTVF176 matched with M2 (M2S1–0), 179KIYHFYFKNDWS190 matched with M3 (M3S1–0), 191RVATKCYNSGGC202 matched with M4 (M4S1–0), 203AMQYVYEPTYYM214 matched with M1 (M1S1–0), and S1-BCD sub-domain residues 646LDVCTKYTIYGF657 matched with M4 (M4S1-BCD). The amino acid residues were colored according to the Zappos scheme that is based on physicochemical properties. *, identical amino acids; :, conserved amino acids.

Because the mAbG3 was found to enhance the PEDV infectivity, it was interesting to identify the epitope of this monoclonal antibody for the future design of a safe PEDV subunit/DNA vaccine. The Ph.D.^™^-12 Phage Display Peptide Library was used as a tool for the identification of the phage peptides that were bound by the mAbG3, and the mAbG3-bound phage peptides were aligned with the PEDV S1 linear sequence to identify the mAbG3-bound S1 sub-domain region, i.e., mAbG3 epitope. After panning the phage library with the mAbG3, the mAbG3-bound phages were propagated; the phage DNAs were isolated, sequenced, and deduced. The deduced amino acid sequences, designated mimotope (M) types, were aligned with monomeric S1 segments of CV777 classical strain (GenBank: AEX92968.1) and PEDV GII variant P70 strain. The results revealed that there were four phage mimotope (M) types (M1-M4) that matched with the residues in the linear S1 subunit, including M1: FFDYMYLPGFAA, M2: SFDWPTHKMFNL, M3: NLYNYMFADLYT, and M4: TDLCHLYNMHGC. The M1 phage mimotope sequence matched with 203AMQYVYEPTYYM214 of S1-0 (designated M1S1–0), M2 sequence matched with 165GITWDNDRVTVF176 of S1-0 (designated M2S1–0), M3 sequence matched with 179KIYHFYFKNDWS190 of S1-0 (designated M3S1–0), and M4 sequence matched with 191RVATKCYNSGGC202 of S1-0 (designated M4S1–0) and 646LDVCTKYTIYGF657 of S1-BCD (designated M4S1-BCD; Figure 2).

### Truncated recombinant S1 polypeptides

**Figure 3:**
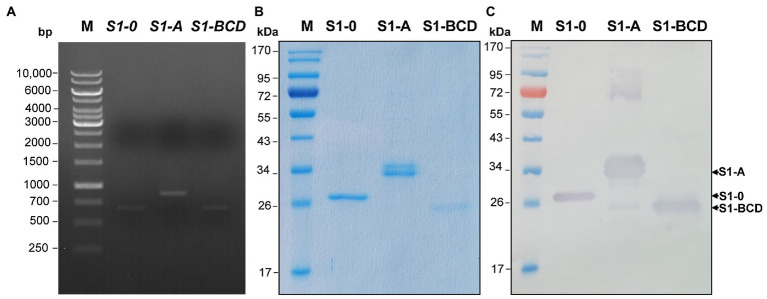
Production of recombinant polypeptides of PEDV S1 subunit (S1-0, S1-A, and S1-BCD). **(A)** PCR amplificons of the DNA sequences coding for the S1-0 (651 bp), S1-A (873 bp), and S1-BCD (660 bp). Lane M, 1 kb DNA ladder. The numbers on the left are DNA sizes in bp. **(B)** SDS-PAGE-separated patterns of the purified S1-0 (26 kDa), S1-A (32 kDa), and S1-BCD (24 kDa) after staining with CBB dye. **(C)** Western blot patterns of the purified S1-0, S1-A, and S1-BCD as detected by mouse anti-6× His antibody. Lanes M of **(B)** and **(C)** are protein molecular mass markers. The numbers on the left of **(B)** and **(C)** are protein molecular masses in kDa.

The truncated polypeptides of the PEDV S1 subunit (S1-0, S1-A, and S1-BCD) were prepared for verification of the S1 regions bound by the mAbG3. Amplicons of the DNA sequences coding for the S1-0, S1-A, and S1-BCD (651, 873, and 660 bp, respectively) are shown in Figure 3A. The recombinant S1 polypeptides with 8× His tag expressed by the transformed NiCo21(DE3) *E. coli* clones were then purified by Ni-NTA affinity resin under denaturing conditions with 8 M urea buffer. The purified polyproteins were then refolded by drop-wise dialysis against PBS. The polypeptides, i.e., S1-0 (residues 1–217; ~26 kDa), S1-A (residues 218–508; ~32 kDa), and S1-BCD (residues 509–728; ~24 kDa) after SDS-PAGE and staining with Coomassie Brilliant Blue G-250 (CBB) dye are shown in Figure 3B. Figure 3C shows Western blot patterns of the SDS-PAGE-separated polypeptides probed with mouse anti-6× His antibody.

### Binding of mAbG3 to recombinant polypeptides of the PEDV S1 subunit

**Figure 4:**
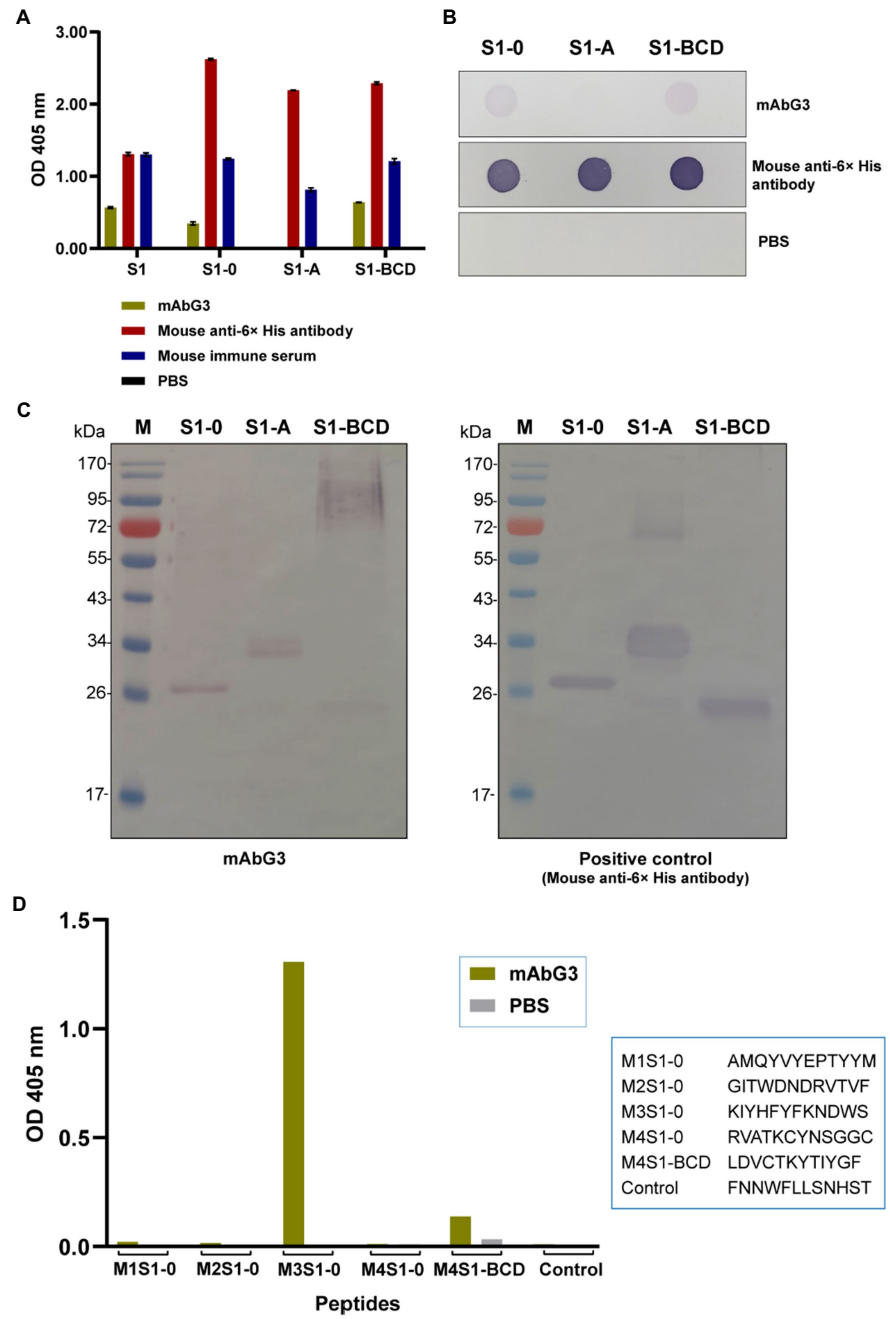
Binding of mAbG3 to recombinant polypeptides S1-0, S1-A, and S1-BCD of the PEDV S1 subunit. **(A)** Indirect ELISA (OD 405 nm) of mAbG3 binding to the full-length recombinant S1 subunit and the S1 polypeptides S1-0, S1-A, and S1-BCD. Mouse anti-6× His antibody and mouse immune serum (1:10,000) served as positive binding controls, and PBS served as a negative binding control. **(B)** Binding of mAbG3 to S1-0, S1-A, and S1-BCD by dot ELISA. Mouse anti-6× His antibody (1:1,000) and PBS served as positive and negative binding controls, respectively. **(C)** Binding of mAbG3 to SDS-PAGE-separated S1-0, S1-A, and S1-BCD by Western blotting (left panel). Mouse anti-6× His antibody (1:1,000) was used as a positive control (right panel). **(D)** Binding of mAbG3 to M1S1–0, M2S1–0, M3S1–0, M4S1–0, M4S1-BCD, and control peptides by peptide binding ELISA. Y-axis, OD 405 nm; X-axis, peptides used as the test antigens; PBS served as control.

The mAbG3 was tested for binding to the purified full-length S1 and S1-0, S1-A, and S1-BCD polypeptides in the indirect ELISA, dot ELISA, and Western blot analysis. The indirect and dot ELISA results showed that the mAbG3 bound to the full-length S1 subunit and the S1-0 and S1-BCD polypeptides but not the S1-A polypeptide (Figures 4A,B). When the mAbG3 was tested for binding to the S1 polypeptides by Western blot analysis under reducing conditions using mouse anti-6× His antibody as positive binding control, the mAbG3 bound to all three S1 sub-domain polypeptides (Figure 4C).

### Verification of the mAbG3 epitope by using peptide binding ELISA

Biotin-labeled M1S1–0, M2S1–0, M3S1–0, M4S1–0, M4S1-BCD, and control peptides were commercially synthesized and used as antigens in the indirect ELISA for testing the mAbG3 binding. In the indirect ELISA process, the synthesized peptides were used to coat separate wells of a microplate, and the coated wells were added with the mAbG3. The mAbG3 yielded a strong binding signal to M3S1–0, a significant signal to the M4S1-BCD, negligible signals to M1S1–0, M2S1–0, and M4S1–0, and no signal to control peptide (Figure 4D).

### Multiple sequence alignment of S1 proteins of different PEDV isolates

**Figure 5:**
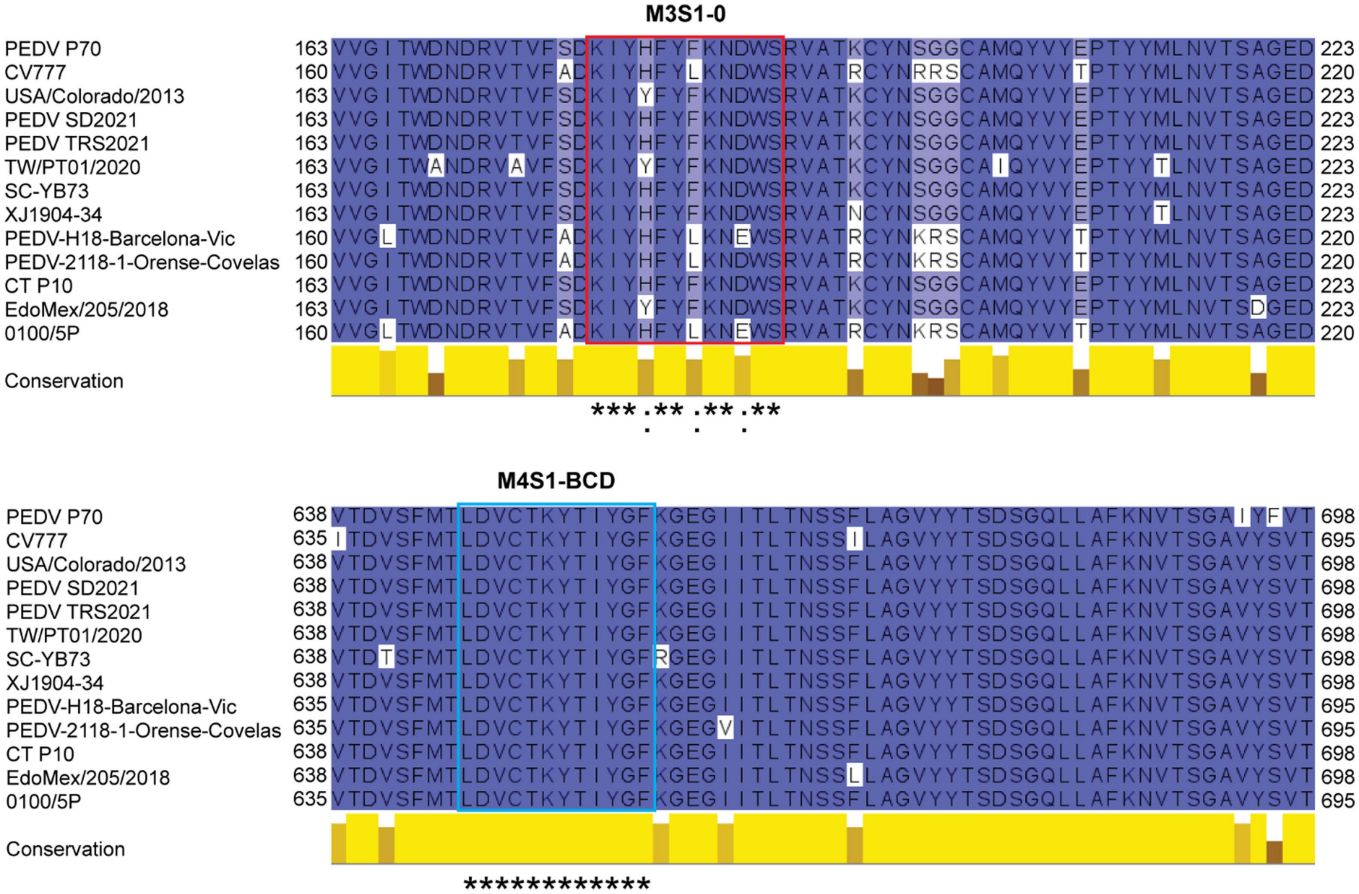
Multiple sequence alignment of amino acid sequences of spike proteins of various PEDV strains including PEDV P70 (a strain that was used in this study), CV777 (classical strain), USA/Colorado/2013 (prototype strain that was used for producing baculovirus-derived S subunit vaccine that caused severe enhancement; GenBank: KF272920.1), PEDV SD2021 (GenBank: UJZ92254.1), PEDV TRS2021 (GenBank: UJZ92268.1), TW/PT01/2020 (GenBank: UOK15705.1), SC-YB73 (GenBank: QNL15619.1), XJ1904-34 (GenBank: UGN13709.1), PEDV-H18-Barcelona-Vic (GenBank: QKV43853.1), PEDV-2118-1-Orense-Covelas (GenBank: QKV43823.1), CT P10 (GenBank: QHB92364.1), EdoMex/205/2018 (GenBank: QQK84868.1), and 0100/5P (GenBank: UAJ21031.1). The M3S1–0 sequences (red box) are highly conserved, and M4S1-BCD sequences (blue box) are identical among these PEDV strains. *, identical amino acids; :, conserved amino acids.

**Figure 6:**
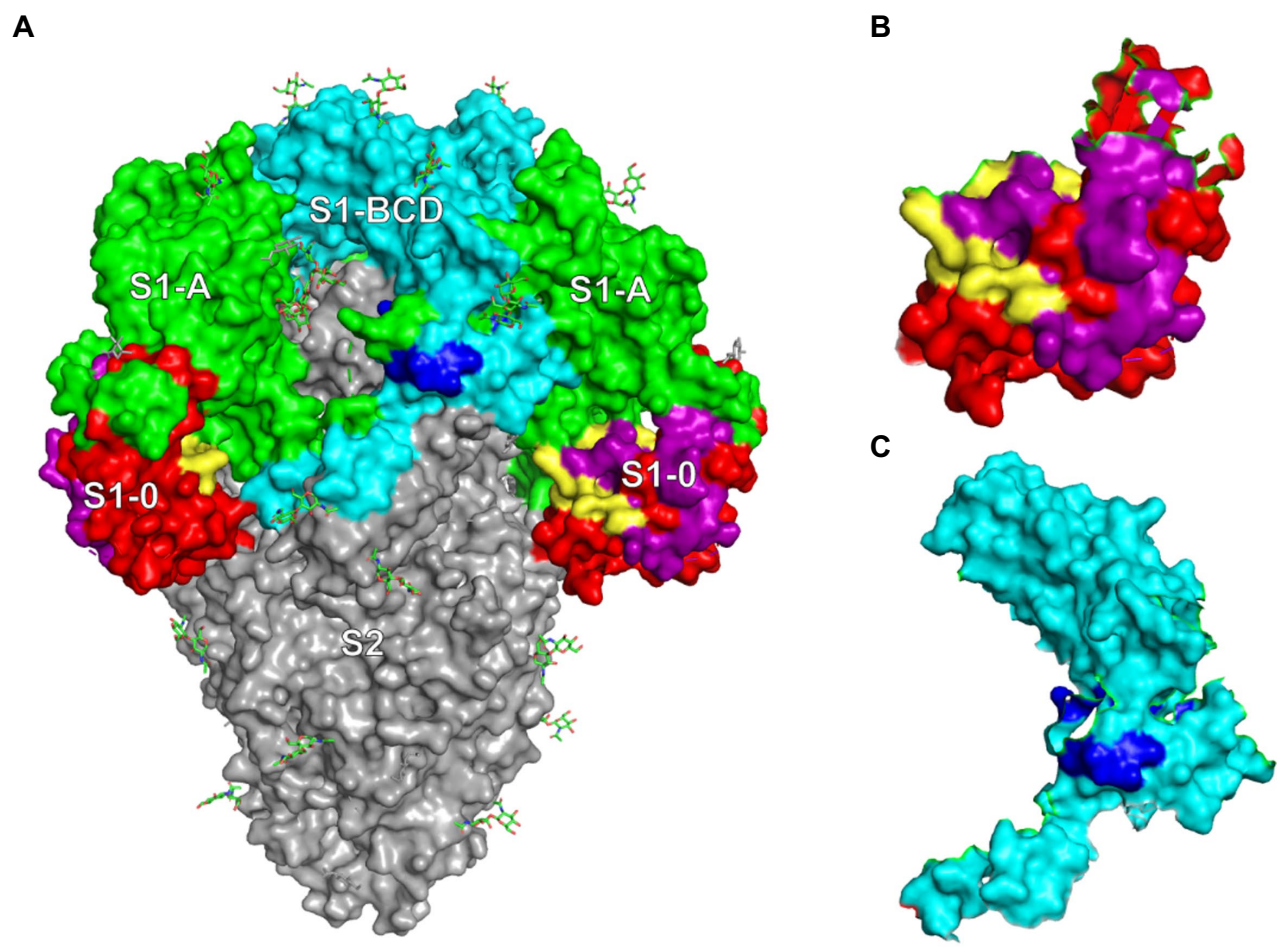
**(A)** PEDV trimeric spike protein (side view) showing locations of S1-0 (red, purple, and yellow), S1-A (green), S1-BCD (cyan and blue), and S2 subunits (gray). **(B)** Monomeric S1-0 showing the regions matched with phage mimotopes M1-M4 (purple and yellow); the yellow area is the M3S1–0. **(C)** S1-BCD monomer (cyan) showing the region bound by the mAbG3 (M4S1-BCD in blue).

The results revealed that the M3S1–0 and M4S1-BCD that were bound by the mAbG3 are highly conserved (Figure 5). Locations of the mAbG3 epitope on the PEDV trimeric S, monomeric S1-0, and monomeric S1-BCD proteins are shown in Figures 6A–C, respectively.

## Discussion

Antibodies are important immunological factors for host defense against virus infections. The antibodies neutralize the virus infectivity by binding to the virus particles and interfering with the virus attachment/binding to the host receptors and/or preventing the uncoating of the viral genome and release into the cytoplasm, thus inhibiting further replication. The antibody-mediated antiviral activities may also involve complement-mediated lysis of the virus particles/virus-infected cells, opsonization of the virus particles for phagocytosis, and antibody-dependent cell-mediated cytotoxicity (ADCC) by cytotoxic T lymphocytes and NK cells (Forthal, 2014). These effective antibodies (may be vaccine-induced, disease convalescing, or passively acquired) mainly target the surface-exposed virus ligands, e.g., spike (S) proteins of coronaviruses, glycoprotein (G) protein of rabies virus, VP proteins of enteroviruses, etc. The efficacy of the antibodies depends on the affinity as well as target accessibility and occupancy (Dowd et al., 2011; Ertl, 2019; Densumite et al., 2021; Edara et al., 2021). It was during the 1960s-1970s that a dark side of the antibodies in virus infection and immunity was recognized. IgG in the avian antiserum mediated immune enhancement of infectivity in rabbitpox virus and Murray Valley encephalitis virus when assayed in the avian cell culture system (Hawkes and Lafferty, 1967). It was also observed that secondary infection with dengue viruses was associated with severe illness along with higher viremia (Libraty et al., 2002; Halstead, 2012). The paradoxical role of antibodies in the dengue infection was found to be caused by sub- or non- neutralizing antibodies which facilitate the virus entry into Fc receptor bearing host cells, e.g., monocytes, macrophages, and dendritic cells, *via* the Fc fragments, which leads to more viral load and hence more clinical severity (Kulkarni, 2020). The phenomenon is currently known as “extrinsic” antibody-dependent enhancement (ADE; Kulkarni, 2020). The extrinsic ADE is mainly mediated by IgG, but IgM and IgA can also induce ADE (Janoff et al., 1995). Currently, several other molecular mechanisms of ADE that aggravate viral diseases have been recognized, including intrinsic ADE which results in heightened virus production by inhibition of type 1 interferon and activation of interleukin-10 biosynthesis, thereby favoring a Th2-type immune response (Narayan and Tripathi, 2020), immune enhancement ADE (Hotez et al., 2020), complement-mediated ADE (Prohászka et al., 1997), and ADE *via* host immune suppression by the intracellular virus (Chareonsirisuthigul et al., 2007). Enhancement of virus entry by non-neutralizing antibodies can be mediated by other mechanisms, e.g., antibody-mediated cross-linking of the spike proteins, which subsequently results in promoting the up-standing/open form of the receptor-binding domain (RBD) of trimeric S protein of SARS-CoV-2 (Liu et al., 2021). For the influenza viruses, the non-neutralizing antibodies promote the increment of hemagglutinin stem flexibility and virus fusion kinetics (Winarski et al., 2019).

One of the many obstacles to vaccine development against virus infections includes the ADE induced by the vaccines at new exposure to the authentic virus. For the PEDV, a baculovirus-expressed recombinant spike protein vaccine enhanced PED symptoms and PEDV replication in challenged immune piglets, although the vaccine provided a high neutralizing antibody titer (Yu et al., 2021). It was speculated that the non-neutralizing antibodies specific to the post-fusion form of the spike protein promoted the PEDV replication in the immune piglets and thus enhanced PED (Yu et al., 2021). However, the enhancing epitope of the S protein has not yet been identified.

In this study, a monoclonal antibody (mAbG3) that bound to the PEDV S1 subunit (Thavorasak et al., 2022) was found to enhance the PEDV infectivity in Vero cell culture. The enhancement was obvious at the high mAbG3 dose (40 μg), while the lower doses (5, 10, and 20 μg) only showed the enhancing trend, but the effect was not different from the infected cells in the medium alone. The higher mAbG3 doses (> 40 μg) were not tested, as the experiment was conducted only to show that the mAbG3 was an enhancing antibody and to find the enhancing epitope. The mAbG3-mediated ADE of the PEDV should not be the extrinsic ADE as the Vero cells lack the Fc receptor. Likewise, the ADE cannot be a result of immune enhancement *via* complement activation and recruitment of inflammatory and immune cells, as there were no complement proteins or inflammatory/immune cells in the *in vitro* assay performed in this study, although this mechanism may occur *in vivo*. More likely, the ADE that is observed when the PEDV-infected cells were incubated with the mAbG3 may be due to intrinsic ADE, i.e., the virus-immune complexes internalized *via* macropinocytosis (Mercer and Helenius, 2009) may modulate innate immune effectors to favor increment of virus replication and release (Narayan and Tripathi, 2020). It was also observed previously that Toll-like receptor 2 (TLR2)-mediated NF-κB activation was inhibited in Vero cells infected with herpesvirus of turkeys (HVT; Yang et al., 2013). Therefore, the PEDV-mAbG3 complexes might inhibit the danger signaling that usually occurs *via* the pathogen recognition receptors (PRRs) and favors the virus replication (Flipse et al., 2016). The mAbG3 may cross-link the spike proteins which eventually results in promoting the up-standing/open form of the receptor-binding domain (RBD) of trimeric S protein of the PEDV, as observed for SARS-CoV-2 (Liu et al., 2021).

Phage Display Peptide Library was used as a biological tool for searching the location of the enhancing epitope of the PEDV S protein. After the bio-panning of peptide phage with the immobilized mAbG3, four phage mimotope types (M1-M4) were obtained. Alignments of the four phage mimotope/peptide sequences with the linear S1 subunit sequences of both GI (classical CV777) and GII (field isolated P70) strains of PEDV found that the S1 sub-domain of both PEDV strains that matched with the phage mimotope sequences was located in the multiple regions of the S1-0 sub-domain, i.e., M1S1–0, M2S1–0, M3S1–0, and M4S1–0, as well as a linear sequence in the S1-BCD sub-domain (M4S1-BCD). The finding was verified by both indirect ELISA and dot ELISA by using the S1 truncated recombinant polypeptides as antigens in the assays and by the peptide binding ELISA. These findings indicate that the epitope of the enhancing mAbG3 is shared by the S1-0 and the S1-BCD sub-domains. In the peptide binding ELISA, the mAbG3 produced negligible ELISA signals to the M1S1–0, M2S1–0, and M4S1–0. Some residues in these three regions may participate in forming the mAbG3 conformational (structural) epitope (their residues were juxtaposed with the M3S1–0 upon the S1 folding to form the conformational epitope but could not be bound by the mAbG3 tight enough to give significant signals in their linear sequences).

There was no mAbG3-bound phage peptide that matched with the sequence of the S1-A sub-domain, although the mAbG3 bound to the S1-A polypeptide by Western blotting. In the phage panning with the immobilized mAbG3, the phage displaying peptide that matched with the S1-A peptide might not be able to compete with the phages that displayed mimotopes for S1-0 and S1-BCD and/or might be due to less population of the former than the latter in the Ph.D.^™^-12 Phage Display Library. The binding test of the mAbG3 with the three truncated S1 subunit polypeptides in Western blot analysis showed that the mAbG3 could bind to the S1-A polypeptide, although the antibody did not bind to the S1-A when tested by both ELISAs. These findings can be explained by the different physicochemical structures of the proteins when they were used as antigens in the assays of different principles (ELISA *versus* Western blotting). The monomeric recombinant S1-A in the free folding structure was coated or dotted onto the supported matrices (well surface of the ELISA plate or nitrocellulose membrane). In these conditions, the S1-A epitope might be hidden in the folded protein and could not be accessible to the mAbG3, thus producing negative ELISA results. In the Western blot analysis, the antigen (S1-A polypeptide) was exposed to reducing agents like 2-mercaptoethanol and SDS; the SDS was also bound to the polypeptide during the electrophoresis. Therefore, the antigen should acquire the stretched (linear) configuration which exposed the mAbG3 recognition site on the blotted membrane, thus giving rise to the antigen–antibody reactive band after adding the mAbG3. This mAbG3-bound region should not be surface-exposed on the trimeric S protein of authentic PEDV and hence should not contribute to the ADE.

The sequence of M4 matched with two sites on the PEDV S1 subunit, i.e., 191RVATKCYNSGGC202 of the S1-0 (M4S1–0) and 646LDVCTKYTIYGF657 of the S1-BCD (M4S1-BCD). When mapping these two regions on the trimeric S protein (Figure 6A), it was found that only the residues 646LDVC649 of the S1-BCD are exposed, while the 650TKYTIYGF657 are obscured underneath. The mAbG3 could access the S1-BCD epitope in the ELISA, as the recombinant S1-BCD used as the antigen was monomeric and thus exposing the epitope. Naturally, only a small portion of the S1-BCD would be exposed on the functionally active trimeric spike protein, while the S1-0191RVATKCYNSGGC202 (M4S1–0) is fully surface-exposed. Binding of the antibody specific to this epitope and the S1-BCD surface-exposed epitope portion could cause extrinsic ADE (enhancement of PEDV entry and increased replication) and immune enhancement ADE (the virus-immune complexes internalized *via* macropinocytosis and modulate innate antivirus activity to favor increment of virus replication and release; Mercer and Helenius, 2009; Narayan and Tripathi, 2020).

Taken all the data together, the enhancing epitope of the PEDV S1 subunit that may cause ADE was found to be located at the C terminus of the S1-0 sub-domain and S1-BCD. Although the exact residues that participate in the epitope formation need further elucidation (such as by co-crystallography), the finding in this study together with the information on the known S1 neutralizing epitopes identified previously (Chang et al., 2002, 2019, 2021; Cruz et al., 2008; Sun et al., 2008; Okda et al., 2017; Ho et al., 2020; Tien et al., 2021; Thavorasak et al., 2022) should be useful in designing a safe PEDV vaccine, i.e., by using a recombinant spike (S) protein whose C-terminal portion of S1-0 and S1-BCD have been deleted and contain only the neutralizing B-cell epitopes along with appropriate T-cell epitopes as immunogens in a subunit protein vaccine or by using the respective DNA counterparts as a DNA vaccine.

## Data availability statement

The original contributions presented in the study are included in the article/Supplementary Material. Further inquiries can be directed to the corresponding author.

## Author contributions

WC, NS, and KT: conceptualization, project administration, and funding acquisition. WC, NS, MC, KG-a, KM, TS, RY, and DN: methodology. WC and TT: validation. WC: formal analysis and data curation. TT, MC, KG-a, KM, TS, RY, DN, and NS-l: investigation. WC, KT, and NS: resources. WC and TT original draft preparation. WC, NS, and TT: reviewing and editing. TT, MC, and KG-a: visualization. WC, NS, KT, MC, KG-a, and KM: supervision. All authors have read and agreed to the published version of the manuscript.

## Funding

This research was funded by the Program Management Unit B (PMU.B), Ministry of Higher Education Science Research and Innovation, Thailand, and Mahidol University. TT received a Research and Researchers for Industries (RRI) scholarship grant (PHD61I0028) from the National Research Council of Thailand (NRCT), Ministry of Higher Education, Research and Innovation, Thailand.

## Conflict of interest

KT was employed by the MORENA Solution Company.

The remaining authors declare that the research was conducted in the absence of any commercial or financial relationships that could be construed as a potential conflict of interest.

## Publisher’s note

All claims expressed in this article are solely those of the authors and do not necessarily represent those of their affiliated organizations, or those of the publisher, the editors and the reviewers. Any product that may be evaluated in this article, or claim that may be made by its manufacturer, is not guaranteed or endorsed by the publisher.

## Footnotes

^1^ https://digitalinsights.qiagen.com/
