# Supplementary material for: Enhancing epitope of PEDV spike protein
Source: Front Microbiol. 2022 Jul 22;13:933249. doi: 10.3389/fmicb.2022.933249 (PMC9355140; doi:10.3389/fmicb.2022.933249)
Supplement: Supplementary file 1 [file Data_Sheet_1.pdf]

Supplementary Materials

Supplementary Table S1 | Oligonucleotide primers used in qRT-PCR (Cho et al., 2012).

Gene	Primer name	Sequence (5′-3′)
PEDV *N* (nucleocapsid gene)	PEDV N F′	CAAGCACTTCTGTTTCCCCGG
	PEDV N R′	ATTGTCACCATAAGCAGCCA
RPLPO (endogenous control; ribosomal protein lateral stalk subunit P0 gene)	RPLPO F′	AGATGCAGCAGATCCGCAT
	RPLPO R′	GGATGGCCTTGCGCA

Reference

Cho, W.K., Kim, H., Choi, Y.J., Yim, N.H., Yang, H.J., and Ma, J.Y. (2012). *Epimedium koreanum* Nakai water extract exhibits antiviral activity against porcine epidermic diarrhea virus *in vitro* and *in vivo*. *Evid. Based Complement. Alternat. Med*. 2012:985151. doi: 10.1155/2012/985151

Supplementary Table S2 | Oligonucleotide primers used in this study for production of truncated recombinant S1 polypeptides of PEDV spike protein.

Gene	Primer name	Sequence (5′-3′)
S1-0	S1-0 F′	GCGGATCCGATGAARTCYTTAAMYTACTTCTGG
	S1-0 R′	GGGCTCGAGTGAACCGCCTCCACCAACATTAAGCATGTAGTAAGTGGG
S1-A	S1-A F′	GCGGATCCGACTAGTGCTGGTGAGGATG
	S1-A R′	GGGCTCGAGTGAACCGCCTCCACCATTAAATGATGGCAAAGTAACAAAAG
S1-BCD	S1-BCD F′	GCGGATCCGGATCATTCTTTTGTTAATATTACTGTCTCTG
	S1-BCD R′	GGGCTCGAGTGAACCGCCTCCACCAGTATTGCTAAACGTGGAGC
